# Long-term effects on function, health-related quality of life and work ability after structured physiotherapy including a workplace intervention. A secondary analysis of a randomised controlled trial (WorkUp) in primary care for patients with neck and/or back pain

**DOI:** 10.1080/02813432.2020.1717081

**Published:** 2020-01-30

**Authors:** Malin H. Forsbrand, Aleksandra Turkiewicz, Ingemar F. Petersson, Charlotte Post Sennehed, Kjerstin Stigmar

**Affiliations:** a Faculty of Medicine, Department of Clinical Sciences Lund, Orthopedics, Lund University, Lund, Sweden;; b Blekinge Centre of Competence, Region Blekinge, Karlskrona, Sweden;; c Clinical Epidemiology Unit, Orthopedics, Clinical Sciences, Lund, Lund University, Lund, Sweden;; d Skåne University Hospital, Lund, Sweden;; e Department of Research and Development, Region Kronoberg, Växjö, Sweden;; f Department of Health Sciences, Physiotherapy, Lund University, Lund, Sweden

**Keywords:** Neck pain, back pain, function, health-related quality of life, work ability, workplace intervention, primary care

## Abstract

**Objective:** To study the long-term effects of a workplace intervention in addition to structured physiotherapy regarding self-reported measures in patients with acute/subacute neck and/or back pain.

**Design:** WorkUp – a cluster-randomised controlled trial in 32 primary care centers in Sweden, from January 2013 through December 2014 (ClinicalTrials.gov ID: NCT02609750).

**Intervention:** Structured physiotherapy with the workplace dialogue ‘Convergence Dialogue Meeting’ (CDM), conducted by the treating physiotherapist as an add-on. Reference group received structured physiotherapy.

**Subjects:** Adults, 18–67 years (mean 43.7, standard deviation (SD) 12.2), 65.3% women with acute/subacute neck and/or back pain who had worked ≥4 weeks past year, considered at risk of sick leave or were on short-term sick leave (≤60 days) were included (*n* = 352).

**Outcome measures:** Self-reported function, functional rating index (FRI), health-related quality of life (EQ-5D-3L) and work ability (Work Ability Score, WAS) at 12 months follow-up.

**Results:** The mean differences in outcomes between intervention and reference group were; −0.76 (95% confidence interval (CI): −2.39, 0.88; FRI), 0.02 (95% CI: −0.04, 0.08; EQ-5D-3L) and −0.05 (95% CI: −0.63, 0.53; WAS). From baseline to 12 months, the intervention group improved function from 46.5 (SD 19.7) to 10.5 (SD 7.3) on FRI; health-related quality of life from 0.53 (SD 0.29) to 0.74 (SD 0.20) on EQ-5D and work ability from 5.7 (SD 2.6) to 7.6 (SD 2.1) on WAS.

**Conclusion:** Despite a clinically relevant improvement over time, there were no significant differences in improvement between groups, thus we conclude that CDM had no effect on self-reported measures in this study.

**Key points**
- In earlier analysis of the primary outcome (work ability measured by absenteeism) in this trial, a positive effect was found when the workplace intervention ‘Convergence Dialogue Meeting’ (CDM) was added to structured physiotherapy for patients with neck or back pain.
- By contrast, in this new analysis of secondary outcomes (self-reported function, health and perceived work ability), there was no added effect of CDM above structured physiotherapy alone, although patients in both the intervention and reference group improved over time.
- The addition of CDM to physiotherapy is therefore justified by its specific effect on behavior (work absence) rather than any effect on clinical outcomes.

## Introduction

Musculoskeletal pain, especially back pain (BP) and neck pain (NP), is very common in the general population [1]. In 2015, BP and NP were the leading global causes of years lived with disability worldwide [2] and these individuals are often seen in primary care [3]. Individuals seeking care for BP consume close to twice as much health care compared with the general population [4]. Most individuals with new episodes of BP recover quickly but recurrence is common and for some, the pain becomes persistent and disabling [5]. Evidence is mounting that BP should be treated more like a long-lasting condition with a variable course rather than unrelated episodes of BP [6]. Individuals with NP and/or BP have higher risk of reporting reduced work ability [7], decreased functional ability [8] and poor health-related quality of life (HRQoL) compared with those without pain [9,10]. The socio-economic consequences in terms of work disability, sick leave and health care costs are large for the individuals and the society [11]. It is therefore important for clinicians to early identify risk factors for work disability [12] and evaluate the effects of early interventions in primary care.

Early interventions and interventions with workplace involvement are important factors in preventing work disability [13] and implementing multi-domain interventions has been reported to reduce duration away from work for individuals with musculoskeletal pain [14]. Improving social and organisational support in the workplace may also be an effective strategy in reducing functional limitation and disability in patients with BP [15]. Vocational rehabilitation is strongly recommended but to be effective, work-focused healthcare and accommodating workplaces must be coordinated [16].

The ‘Convergence Dialogue Meetings’ (CDM) is a workplace dialogue between the patient, the health care provider and the employer, originally developed for patients on long-term sick leave due to burnout syndromes [17]. The CDM model consists of a three-step structured interview where the patient, the health care provider and the employer meet and together identify the needs for workplace adjustments and the aim is to find concrete suggestions and actions to support and maintain work ability or if sick-listed, facilitate return-to-work. In the WorkUp trial [18], the CDM has, in addition to structured physiotherapy, shown to increase the odds for having work ability at one-year follow-up (odd ratio (OR) 1.85, 95% confidence interval (CI) 1.01–3.38; work ability defined as working at least four consecutive weeks at one year follow-up) for patients with NP and/or BP. The CDM has also shown to be a cost-effective intervention in primary care, both from a societal and healthcare perspective [19]. The CDM has not been evaluated from the patient’s perspective in terms of self-reported data on function, HRQoL and work ability. The objective of the current study was therefore to study the long-term effects (one- year) of the workplace dialogue CDM, in addition to structured physiotherapy, regarding secondary endpoints in the WorkUp trial: self-reported function, HRQoL and work ability in patients with acute/subacute NP and/or BP in primary care. Our hypothesis was that patients in both groups would improve over time regarding all three outcomes. Further, we expected work ability to improve more in the intervention group.

## Material and methods

We conducted a prospective pairwise cluster-randomised controlled trial in ordinary Swedish primary care with 12 months follow-up. Inclusion of patients was performed during January 2013 to December 2014 (ClinicalTrials.gov ID: NCT02609750) [18]. In this study we analyse the secondary outcomes in the WorkUp trial.

A total of 32 public and private primary care centers in southern Sweden, linked to 20 primary care rehabilitation units (including physiotherapists), took part in the trial. The rehabilitation units were matched in pairs, based on similar size (registered population and community size), health care need (adjusted clinical groups (ACG) and socioeconomic standard (care need index (CNI) and then randomised into 10 intervention units and 10 reference rehabilitation units. Each rehabilitation unit was either an intervention unit or a reference unit, never mixed. The patients and the rehabilitation units staff (including the physiotherapists) were therefore not blinded to allocation. A more detailed description of the randomisation process and the power analysis for the main confirmatory outcome work ability can be found elsewhere [18].

### Study groups

Adults, in working age (18–67 years), who applied for physiotherapy by direct access in ordinary Swedish primary care due to an episode of acute/subacute (<12 weeks) NP and/or BP were eligible for inclusion. It could be either a first episode or a recurrent episode of NP and/or BP, after a period of at least three months of no substantial pain. After screening and informed consent the patients were asked to participate in the study if they had worked at least four consecutive weeks past year, had no more than 60 days of sick leave or were not currently on sick leave, but still considered at risk of sick leave, by scoring ≥40 points on the short form of the Örebro Musculoskeletal Pain Screening Questionnaire (ÖMPSQ-short) [20]. Patients with full time disability pension, addiction diagnose, on-going medical treatment for an acute disease, pregnancy or unable to understand the Swedish language were excluded. After information in verbal and writing, 352 individuals were included in the trial after informed consent; 146 in the intervention group and 206 included in the reference group.

### Procedure

At the first visit, the patients were examined by a physiotherapist. Signs of serious medical conditions (‘red flags’) and psychosocial risk factors (‘yellow flags’) were considered. If there were medical conditions in urgent need for medical care or examination, patients were referred to a doctor without delay. The patients completed a questionnaire with self-reported measures which also were completed after three-, six- and 12 months. The physiotherapy treatment was evidence-based and structured including return visits to the physiotherapist at three-, six- and 12-months follow-up. The treatment was individualised in terms of content and duration according to each patient’s condition. Based on patient´s needs and clinical assessments also other health care professionals could be engaged, i.e. a doctor, psychologist or an occupational therapist. Further referral to these professionals was based on ordinary clinical assessments, such as red and yellow flags. Within the WorkUp trial, but not for the aim of this study, patients also received weakly short text messages during the one year [18] and were asked to complete some clinician reported measures at baseline and at follow-ups (data to be published).

### Intervention

In addition to the structured physiotherapy treatment, patients in the intervention group were offered CDM by their treating physiotherapist. The physiotherapist started CDM by an individual interview with the patient and the physiotherapist asked the patient about his/her consent for contacting the employer. In the second step, the employer was invited to talk to the physiotherapist, either in person or by phone. In the third step, the patient and the employer were invited to a convergence meeting together with the physiotherapist. The interviews were structured with questions that focused on NP and/or BP in relation to work and on possible or already conducted workplace adjustments. The aim of the CDM was to strengthen the patient’s work ability and to support him/her to stay at work or return to work. The last meeting with the patient, the employer and the physiotherapist ended up in a written plan of action with suggested workplace adjustments and changes to the patient’s daily life. The plan could also include contacts with other stakeholders [18]. The plan of action was then followed up at the return visits to the physiotherapist.

### Self-reported baseline and outcome measures

The baseline questionnaire included type of treatment received (intervention or reference), age, sex, marital status, educational level, if born in Sweden, if on sick leave, International Classification of Diseases-10 diagnose, employment, symptoms of anxiety and/or depression (yes: ≥8 points on hospital anxiety and depression scale (HADS) [21], symptoms of exhaustion (no, moderate or pronounced exhaustion, according to the self-rating of stress-related exhaustion disorder (s-ED) [22]. We defined persons with any percentage of sick leave as sick listed. We did not ask for the reason of sick leave.

### Function

Function was measured with the functional rating index (FRI) which is an instrument designed to measure the subjective perception of functional status and pain in patients with spinal pain [23]. Using a five-point scale for each item, the patient ranks his/her perceived different functions and activities, in relation to daily life. The total score is calculated as recommended by Feise et al. [23] with the range of scores from 0 to 100% disability. The higher the number, the higher perceived disability and pain.

### HRQoL

HRQoL was measured with the EuroQol five-dimension (EQ-5D, 3 L) questionnaire [24]. We used the EQ-index which covers five dimensions of health: mobility, self-care, usual activities, pain/discomfort and anxiety/depression. Each dimension receives a score of one to three, based on three levels of severity: ‘no problems, moderate problems and severe problems’. We used the UK tariff to derive scores ranging from −0.594 to 1.0 where lower EQ-5D values reflect lower HRQoL.

### Work ability

To measure work ability we used the Work Ability Score (WAS) which is the first single-item question (‘current work ability compared with the lifetime best’) from the widely used work ability index (WAI) [25]. The WAS is a good alternative to the complete WAI and a reliable measure for assessing the status and progress of work ability [26,27]. WAS ranges from 0 to 10 where the patient rank his/her current work ability from 0 representing ‘cannot work at all right now’ to 10 representing ‘my work ability as at its best right now’. The WAS classify work ability using the same type of categorisation as the whole WAI, namely: poor (0–5 points), moderate (6,7), good (8,9) and excellent work ability (10).

### Statistical methods

Descriptive statistics on the study sample is presented by randomisation group with means and standard deviations or frequencies and percentages as appropriate. We strived to maintain the intention-to-treat approach. All persons with at least one measurement (at baseline, three, six or 12 months) were included in the analyses of WAS and EQ-5D. For the analysis of FRI, all persons with baseline value and at least one follow-up value were included. We used a linear mixed effect regression model with the rehabilitation unit and the individual as random effects, individuals were nested within rehabilitation units. The treatment group, follow-up time (as categorical variable) and their interaction were included as fixed effects. The estimates for the interaction effect between treatment group and follow-up time represent the between group difference between the two treatments. The between group difference at 12 months was the primary outcome in these analyses. The regression model was adjusted for the baseline value of the respective outcome variable and for age, sex and if on sick leave to account for a possible imbalance between the treatment groups. We performed model diagnostic of all models to check if the underlying assumptions were fulfilled. We used the ‘margins’ command (as implemented in Stata) to estimate the mean values in each treatment group and follow-up occasions presented in the figures. In a sensitivity analysis we repeated the above estimation using the above mentioned regression model additionally adjusted for (a) symptoms of anxiety and/or depression using HADS group (cut-off ≥8) [21] (three categories) or (b) symptoms of exhaustion (yes or no) using the s-ED [22], as measured at baseline. All estimates are given with 95% CI. We used Stata version 15 for statistical analyses (StataCorp, College Station, TX).

## Results

We included a total of 352 individuals (mean age 43.7 [SD 12.2] years, 65.3% women) in the study, 146 in the intervention group and 206 in the reference group. The intervention and reference groups were comparable at baseline (Table 1). At 12 months follow-up, there were 115 patients (79%) in the intervention group and 171 patients (83%) in the reference group who had answered the self-reported questionnaires. Losses and reasons for exclusions of patients after randomisation are presented in the confirmatory study of WorkUp [18].

**Table 1.** Baseline data for patients included in WorkUp, *n* = 352.^a^

	Reference group (n = 206)		Intervention group (n = 146)	
	*n*	%	*n*	%
Men	68	33.0	54	37.0
Women	138	67.0	92	63.0
Age (mean, SD)	43.7	12.6	43.8	11.7
Marital status				
Married/cohabitation	157	77.0	112	76.7
Single	47	22.8	34	23.3
Missing	2			
Education				
Primary school	14	6.8	16	11.0
Upper sec school 2–3 years	107	51.9	69	47.3
University ≥3 years	49	23.8	28	19.2
Other	35	16.9	33	22.6
Born in Sweden				
Yes	173	84.0	132	90.4
No	32	15.5	14	9.6
Missing	1			
Diagnoses				
Cervicobrachial syndrome^b^	49	23.8	27	18.5
Cervical and lumbar syndrome^c^	12	5.8	9	6.2
Lumbago-ischias^d^	140	68.0	102	69.9
Myalgia^e^	5	2.4	8	5.5
Employed				
Yes	194	94.6	142	97.3
No	11	5.4	4	2.7
Missing	1			
Sick leave				
Yes	74	35.9	51	34.9
If yes, 100% sick leave	62	83.8	40	78.4
Missing	1		2	
Anxiety and depression (HADS 0-21 p)				
HADS-A <8 points and HADS-D <8 p	128	62.1	95	65.1
HADS-A ≥8 points or HADS-D ≥8 p	48	23.3	40	27.4
HADS-A ≥8 points and HADS-D ≥8 p	29	14.1	10	6.8
Missing	1		1	
Exhaustion (s-ED)				
Yes	54	26.2	38	26
If yes, moderate s-ED	28	13.6	25	17.1
If yes, pronounced s-ED	26	12.6	13	8.9
Missing	11		10	

HADS: hospital anxiety and depression scale; HADS-A: Anxiety subscale of HADS;

HADS-D: Depression subscale of HADS; s-ED: self-rating of stress-related exhaustion disorder.

a The numbers are frequencies (percentages) unless stated otherwise.

b M530, M531 and M542.

c Combination of cervicobrachial syndrome and lumbago-ischias.

d M543, M544, M545 and M546.

e M791.

### Between group comparisons

The mean differences in outcomes between the intervention and the reference group (adjusted for age, sex, if on sick leave and the baseline value of the outcome) for all three outcomes were small and not statistically significant (Table 2). The mean differences in outcomes between the intervention and the reference group after 12 months were for function: -0.76 (95% CI: -2.39, 0.88)(FRI), for health-related quality of life: 0.02 (95% CI: -0.04, 0.08)(EQ-5D) and for work ability: -0.05 (95% CI: -0.63, 0.53)(WAS) (Table 2). The results were similar in all sensitivity analyses (Table 2).

**Table 2.** The mean difference in function, HRQoL and work ability between the intervention and the reference group.^a^

	Unadjusted	Adjusted^b^	Adjusted^b^ + HADS	Adjusted^b^ + Exhaustion
	Mean difference [95% CI]	Mean difference [95% CI]	Mean difference [95% CI]	Mean difference [95% CI]
Function (FRI)				
3 months follow-up	−2.17 [−4.42,0.09]	0.28 [−1.80,2.36]	0.37 [−1.64,2.39]	−0.36 [−2.51,1.78]
6 months follow-up	2.16 [−0.83,5.16]	−0.42 [−2.02,1.18]	−0.42 [−2.02,1.19]	−0.06 [−1.71,1.58]
12 months follow-up	1.97 [−1.08,5.02]	−0.76 [−2.39,0.88]	−0.77 [−2.41,0.87]	−0.55 [−2.23,1.14]
HRQoL (EQ-5D)				
3 months follow-up	−0.05 [−0.11,0.02]	−0.05 [−0.11,0.01]	−0.05 [−0.11,0.01]	−0.05 [−0.11,0.02]
6 months follow-up	−0.00 [−0.06,0.06]	−0.01 [−0.07,0.06]	−0.01 [−0.07,0.06]	−0.02 [−0.08,0.05]
12 months follow-up	0.02 [−0.04,0.08]	0.02 [−0.04,0.08]	0.02 [−0.04,0.08]	0.01 [−0.05,0.08]
Work ability (WAS)				
3 months follow-up	−0.66 [−1.23, −0.08]	−0.69 [−1.26, −0.11]	−0.68 [−1.26, −0.11]	−0.66 [−1.25, −0.07]
6 months follow-up	−0.23 [−0.80,0.34]	−0.27 [−0.84,0.30]	−0.27 [−0.84,0.30]	−0.35 [-0.94,0.23]
12 months follow-up	−0.01 [−0.58,0.57]	−0.05 [−0.63,0.53]	−0.04 [−0.62,0.54]	−0.15 [−0.74,0.45]

a The differences are presented at three, six and 12 months, respectively.

b Adjusted for age, sex, if on sick leave at baseline and the baseline value of the outcome. HADS: Hospital Anxiety and Depression Scale; Exhaustion: self-rating of stress-related exhaustion disorder (s-ED); HRQoL: health-related quality of life; FRI: Functional Rating Index (0 to 100% disability); EQ-5D: EuroQol five-dimension (−0.594 to 1.0 where 1 correspond to full health); WAS: work ability score (0 to 10 from 0 representing ‘cannot work at all right now’ to 10 representing ‘my work ability as at its best right now’).

### Changes over time

**Figure 1.**
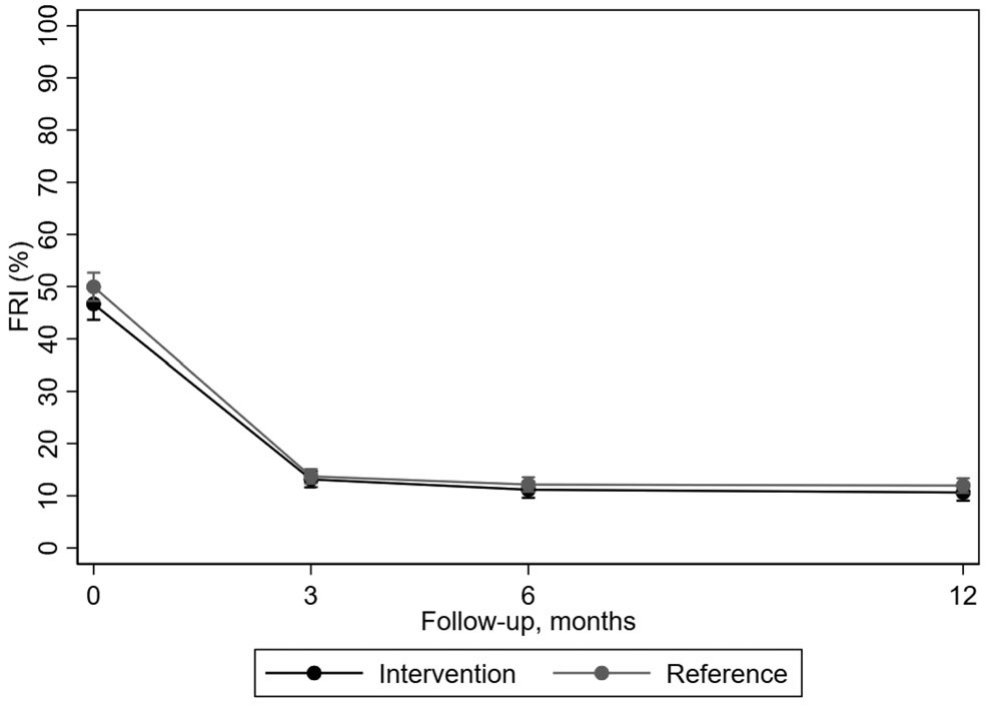
Mean outcome per treatment group over follow-up time with 95% confidence interval estimated by the regression model regarding function as measured by FRI.

**Figure 2.**
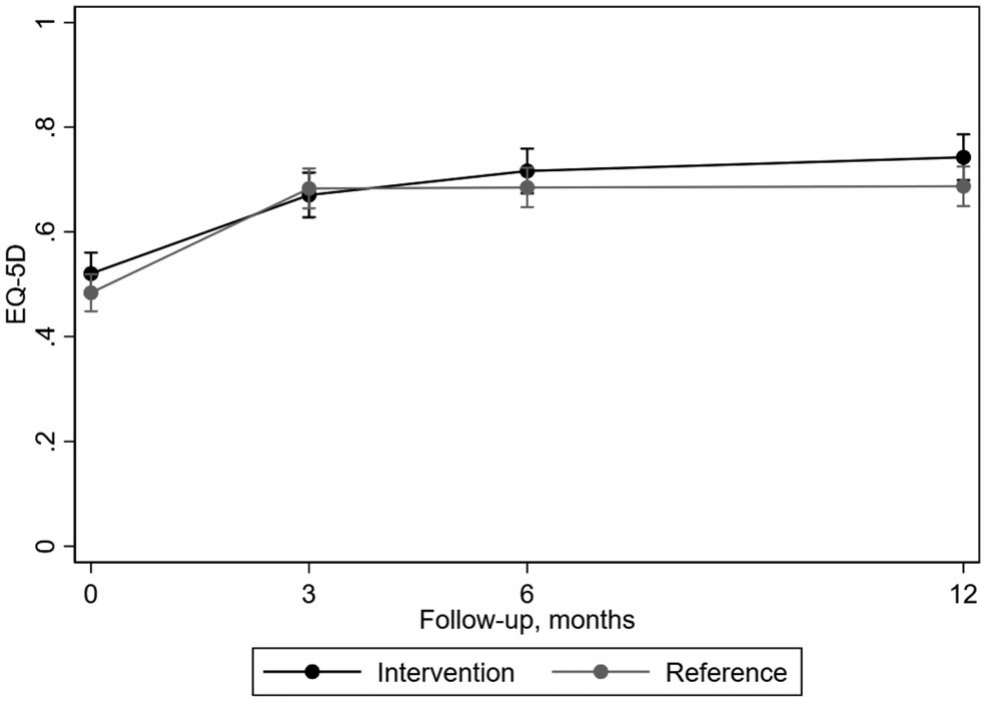
Mean outcome per treatment group over follow-up time with 95% confidence interval estimated by the regression model regarding health-related quality of life as measured by EQ-5D.

**Figure 3.**
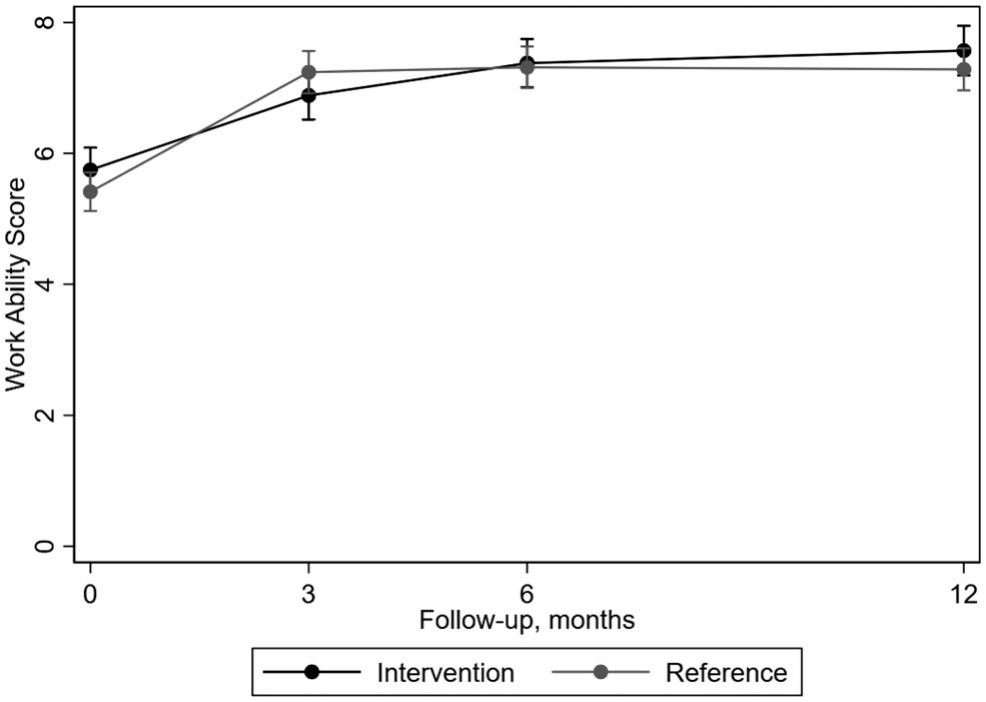
Mean outcome per treatment group over follow-up time with 95% confidence interval estimated by the regression model regarding work ability as measured by work ability score (WAS).

Improvement was observed within both the intervention and the reference group regarding all outcome measures after 12 months (Figures 1–3; Supplementary material). For function, the improvement was most apparent the first three months (Figure 1) and overall, disability decreased in the intervention group between baseline and 12 months from 46.5 (SD 19.7) to 10.5 (SD 7.3) and in the reference group from 49.8 (SD 18.7) to 11.7 (SD 8.2) on FRI.

Health-related quality of life improved during follow-up time (Figure 2). From baseline to 12 months, the intervention group improved from 0.53 (SD 0.29) to 0.74 (SD 0.20) and the reference group from 0.49 (SD 0.30) to 0.69 (SD 0.27) on EQ-5D.

For work ability, patients in both groups improved during the follow-up time, also most apparent between baseline and three months (Figure 3). From baseline to 12 months follow-up, the intervention group improved from 5.7 (SD 2.6) to 7.6 (SD 2.1) and the reference group improved from 5.4 (SD 2.9) to 7.3 (SD 2.4) on WAS.

## Discussions

In this study we included patients in primary care with NP and/or BP, at risk for work disability. The aim was to study what impact a workplace dialogue (CDM) as an add-on to structured physiotherapy, had on self-reported measures. The results show that there were no effects of CDM, as an add-on to structured physiotherapy, on self-reported function, health-related quality of life and work ability at 12 months follow-up. As expected, all self-reported outcomes improved over time in both the intervention and the reference group.

### Strengths and weaknesses of the study

The main strength of this study was inclusion of patients recruited from a large number of primary care settings (32) linked to 20 different rehabilitation units. The implementation was pragmatic and closely linked to the daily clinical practice within primary care. There was only a low risk of ‘contamination’ between rehabilitation units as the units were either an intervention or a reference unit, never mixed. Further strength is the utilisation of a mixed model in the analysis, which enabled us to include all individuals with at least one non-missing value thus minimising potential selection bias and preserving the balance created by the randomisation process. This model also accounts for the missing data during follow-up under the missing at random assumption. Since the CDM was originally developed for patients due to burnout [17], in a sensitivity analysis we chose to adjust for anxiety/depression and exhaustion at baseline. After adjustments, the results did not change substantially, further strengthening our conclusions. A weakness is that we did not record the number of eligible and non-consenting patients, or the reason for this and thus we are careful about the generalisability of the findings. More patients were recruited in the reference group compared to the intervention group (206 vs. 146) and there may have been patients who were eligible for the study but due to different reasons never were asked to participate. However, we have no reason to believe that this was occurring systematically or very often or that the selection of individuals was different in the two arms of the trial. EQ-5D is known not to follow normal distribution. However, we chose to analyse it by using linear regression models and reporting the mean differences as this is a straightforward approach. At inclusion, we used a lower cutoff for ÖMPSQ-short [20] (≥40 points instead of ≥50 points) because we wanted to include patients at risk for long-standing work disability and patients who were at an early stage in their rehabilitation process. Therefore, these patients were found to be clinically relevant for treatment in primary care [18].

### Findings in relation to other studies

In this study, the estimated difference in experience of work ability, measured with WAS at 12 months follow-up, does not support a clinically relevant benefit of added CDM at 12 months follow-up. In the study on confirmatory outcome of the WorkUp trial, we found that the added workplace dialogue CDM was effective compared to structured physiotherapy in reducing work absenteeism at 12 months later [18]. During the follow-up period, work ability was reported with a short text messages on a weekly basis. Patients were asked to answer with a number (0–7), how many days last week, they had been absent from work. The outcome work ability in the previous analysis was defined as working at least four consecutive weeks at follow-up [18]. In this study on secondary outcomes, work ability was measured with WAS as a point-prevalence. WAS captures how the individual experiences his or her work ability at a certain time, but do not need to be associated with work absenteeism. An individual can experience a decreased work ability, but not necessarily having decreased work productivity or being absent from work. There is a range of different ways of assessing work ability and there is no instrument that covers all aspects [28]. Work ability can be seen as ‘a continuum’ where work ability is acknowledged as a dynamic process that changes over time depending on different supportive and/or destructive factors during life [29] and we conclude that the two different measurements of work ability used in the WorkUp trial must not be in concordance with each other as they are measuring different aspects of work ability.

In the present study on secondary outcomes, self-reported work ability did not improve more in the intervention group. Similar results were found in a recent review where work interventions had impact on sick leave but not on self-reported outcomes [30]. This can be an indication of presenteeism; i.e. that patients may experience decreased work ability, even though they are still at work. Brouwer et al showed that >7% of employees in a Dutch firm were working with health problems indicating that productivity losses without absence is quite a common problem [31]. As not all productivity losses are quantitatively expressible (i.e. by sick leave), a quality indication as the WAS may provide valuable additional information about patients work ability. The WAS has shown to be predictive for future disability [27,32] and a planned register study within the WorkUp trial will further study sick leave rates at three years follow-up.

Improvement over time was observed within both the intervention and the reference group regarding all outcome measures after 12 months. Both groups received structured physiotherapy including follow-ups to the treating physiotherapist at three, six and 12 months as well as text messages every week during the one year [18]. We believe that this was a more comprehensive treatment than treatment as usual, and this may have an impact on the almost similar improvements in both groups. As this study did not include a ‘non-treated’ control group we can’t rule out that the improvement in outcomes were the result of either regression to the mean, placebo or other contextual factors [33].

The improvement concerning work ability, resulted in change of WAS category as both groups improved on average from ‘poor’ work ability at baseline to ‘moderate’ work ability at 12 months or from ‘moderate to good’ (if calculated on medians). As a change in the WAS has been validated to show a change in the entire WAI this change must be seen as a clinically relevant improvement [27].

Also function improved in both the reference and the intervention group which is in line with the received physiotherapy treatment that pay much attention to functional limitations. Functional limitations are an important component for having decreased work ability. For example, a recent study reported that functional limitations can have an impact on whether the patients with musculoskeletal pain are on sick leave or not [34].

Most patients in the study population (96%) were employed and patients in both groups improved in HRQoL on average from 0.5 on EQ-5D at baseline to 0.7 on EQ-5D at 12 months. There were no statistically significant differences between groups at 12 months but most patients (85%) reached a score of ≥0.6 on EQ-5D at 12 months which is a proposed limit for having enough capacity to work for patients with BP and NP [35].

## Conclusions

Using self-reported outcomes are increasingly recognised as valuable tools in clinical trials of early interventions by adding unique information about patients benefits of an intervention [36]. In this study we found no effects of the CDM, as an add-on to structured physiotherapy, on self-reported measures at 12 months follow-up. Patients in the intervention and the reference group improved function, health-related quality of life and work ability over time. Although we found no impact of CDM on patient self-report measures in this study, our earlier analysis of the primary outcome of the trial (actual absence from work) showed a positive effect from adding CDM to structured physiotherapy. This finding from the earlier primary outcome analysis together with the cost-effectiveness, must be enough to justify the introduction of the CDM more widely.

## Supplementary Material

Supplemental Material

## References

[1] Bergman S, Herrstrom P, Hogstrom K, et al. Chronic musculoskeletal pain, prevalence rates, and sociodemographic associations in a Swedish population study. J Rheumatol. 2001;28:1369–1377. 11409133

[2] Vos T, Allen C, Arora M, et al. Global, regional, and national incidence, prevalence, and years lived with disability for 310 diseases and injuries, 1990–2015: a systematic analysis for the Global Burden of Disease Study 2015. The Lancet. 2016;388:1545–1602. 10.1016/S0140-6736(16)31678-6 PMC5055577 27733282

[3] Jordan KP, Joud A, Bergknut C, et al. International comparisons of the consultation prevalence of musculoskeletal conditions using population-based healthcare data from England and Sweden. Ann Rheum Dis. 2014;73:212–218. 23345602 10.1136/annrheumdis-2012-202634 PMC3888586

[4] Joud A, Petersson IF, Englund M. Low back pain: epidemiology of consultations. Arthritis Care Res. 2012;64:1084–1088. 10.1002/acr.21642 22337573

[5] Dunn KM, Hestbaek L, Cassidy JD. Low back pain across the life course. Best Pract Res Clin Rheumatol. 2013;27:591–600. 24315141 10.1016/j.berh.2013.09.007

[6] Hartvigsen J, Hancock MJ, Kongsted A, et al. What low back pain is and why we need to pay attention. Lancet. 2018;391:2356–2367. 29573870 10.1016/S0140-6736(18)30480-X

[7] Lindegard A, Larsman P, Hadzibajramovic, Jr. E, et al. The influence of perceived stress and musculoskeletal pain on work performance and work ability in Swedish health care workers. Int Arch Occup Environ Health. 2014;87:373–379. 23609321 10.1007/s00420-013-0875-8 PMC3996278

[8] Heitz CA, Hilfiker R, Bachmann LM, et al. Comparison of risk factors predicting return to work between patients with subacute and chronic non-specific low back pain: systematic review. Eur Spine J. 2009;18:1829–1835. 19565277 10.1007/s00586-009-1083-9 PMC2899435

[9] Nolet PS, Cote P, Kristman VL, et al. Is neck pain associated with worse health-related quality of life 6 months later? A population-based cohort study. Spine J. 2015;15:675–684. 25499207 10.1016/j.spinee.2014.12.009

[10] Nolet PS, Kristman VL, Cote P, et al. Is low back pain associated with worse health-related quality of life 6 months later? Eur Spine J. 2015;24:458–466. 25391622 10.1007/s00586-014-3649-4

[11] Gustavsson A, Bjorkman J, Ljungcrantz C, et al. Socio-economic burden of patients with a diagnosis related to chronic pain–register data of 840,000 Swedish patients. Eur J Pain. 2012;16:289–299. 22323381 10.1016/j.ejpain.2011.07.006

[12] Schaafsma FG, Anema JR, van der Beek AJ. Back pain: prevention and management in the workplace. Baillieres Best Pract Res Clin Rheumatol. 2015;29:483–494. 10.1016/j.berh.2015.04.028 26612243

[13] Gabbay M, Taylor L, Sheppard L, et al. NICE guidance on long-term sickness and incapacity. Br J Gen Pract. 2011;61:e118–24. 21375894 10.3399/bjgp11X561221 PMC3047344

[14] Cullen KL, Irvin E, Collie A, et al. Effectiveness of workplace interventions in return-to-work for musculoskeletal, pain-related and mental health conditions: an update of the evidence and messages for practitioners. J Occup Rehabil. 2018; 28:1–15. 28224415 10.1007/s10926-016-9690-x PMC5820404

[15] Shaw WS, Campbell P, Nelson CC, et al. Effects of workplace, family and cultural influences on low back pain: what opportunities exist to address social factors in general consultations? Best Pract Res Clin Rheumatol. 2013;27:637–648. 24315145 10.1016/j.berh.2013.09.012

[16] Waddell G, Burton AK, Kendall NA. Vocational rehabilitation–what works, for whom, and when? (Report for the Vocational Rehabilitation Task Group). London: TSO; 2008.

[17] Karlson B, Jonsson P, Palsson B, et al. Return to work after a workplace-oriented intervention for patients on sick-leave for burnout–a prospective controlled study. BMC Public Health. 2010;10:301. 20515510 10.1186/1471-2458-10-301 PMC2894773

[18] Sennehed CP, Holmberg S, Axen I, et al. Early workplace dialogue in physiotherapy practice improved work ability at 1-year follow-up-WorkUp, a randomised controlled trial in primary care. Pain. 2018;159:1456–1464. 29554017 10.1097/j.pain.0000000000001216 PMC6085128

[19] Saha S, Grahn B, Gerdtham UG, et al. Structured physiotherapy including a work place intervention for patients with neck and/or back pain in primary care: an economic evaluation. Eur J Health Econ. 2019; 20:317–327. 30171489 10.1007/s10198-018-1003-1 PMC6438933

[20] Linton SJ, Nicholas M, MacDonald S. Development of a short form of the Orebro musculoskeletal pain screening questionnaire. Spine. 2011;36:1891–1895. 21192286 10.1097/BRS.0b013e3181f8f775

[21] Bjelland I, Dahl AA, Haug TT, et al. The validity of the hospital anxiety and depression scale: an updated literature review. J Psychosom Res. 2002;52:69–77. 11832252 10.1016/s0022-3999(01)00296-3

[22] Glise K, Hadzibajramovic E, Jonsdottir I, et al. Self-reported exhaustion: a possible indicator of reduced work ability and increased risk of sickness absence among human service workers. Int Arch Occup Environ Health. 2010;83:511–520. 19943058 10.1007/s00420-009-0490-x

[23] Feise RJ, Michael Menke J. Functional rating index: a new valid and reliable instrument to measure the magnitude of clinical change in spinal conditions. [Erratum appears in Spine 2001 Mar 1;26(5):596]. Spine. 2001;26:78–86. 11148650 10.1097/00007632-200101010-00015

[24] Rabin R, de Charro F. EQ-5D: a measure of health status from the EuroQol Group. Ann Med. 2001;33:337–343. 11491192 10.3109/07853890109002087

[25] Tuomi K, Ilmarinen J, Jahkola A, et al. Work ability index. Helsinki: Finnish Institute of Occupational Health; 1998.

[26] El Fassi M, Bocquet V, Majery N, et al. Work ability assessment in a worker population: comparison and determinants of work ability index and work ability score. BMC Public Health. 2013;13:305. 23565883 10.1186/1471-2458-13-305 PMC3637198

[27] Ahlstrom L, Grimby-Ekman A, Hagberg M, et al. The work ability index and single-item question: associations with sick leave, symptoms, and health–a prospective study of women on long-term sick leave. Scand J Work Environ Health. 2010;36:404–412. 20372766 10.5271/sjweh.2917

[28] Fadyl JK, McPherson KM, Schluter PJ, Turner-Stokes L. Factors contributing to work-ability for injured workers: literature review and comparison with available measures. Disabil Rehabil. 2010;32:1173–1183. 20170279 10.3109/09638281003653302

[29] Lindberg P. The work ability continuum: epidemiological studies of factors promoting sustainable work ability [thesis]. Stockholm: Department of Clinical Neuroscience; 2006.

[30] van Vilsteren M, van Oostrom SH, de Vet HC, et al. Workplace interventions to prevent work disability in workers on sick leave. Cochrane Database Syst Rev. 2015. CD006955. 26436959 10.1002/14651858.CD006955.pub3 PMC9297123

[31] Brouwer WB, Koopmanschap MA, Rutten FF. Productivity losses without absence: measurement validation and empirical evidence. Health Policy. 1999;48:13–27. 10539583 10.1016/s0168-8510(99)00028-7

[32] Kinnunen U, Natti J. Work ability score and future work ability as predictors of register-based disability pension and long-term sickness absence: A three-year follow-up study. Scand J Public Health. 2018;46:321–330. 29212430 10.1177/1403494817745190

[33] Englund M. Bout of the corner men and not the boxers? Contextual effects flex their muscles. Ann Rheum Dis. 2018;77:159–161. 28735281 10.1136/annrheumdis-2017-211664

[34] Stigmar KG, Petersson IF, Joud A, et al. Promoting work ability in a structured national rehabilitation program in patients with musculoskeletal disorders: outcomes and predictors in a prospective cohort study. BMC Musculoskelet Disord. 2013;14:57. 23384339 10.1186/1471-2474-14-57 PMC3626929

[35] Hansson E, Hansson T, Jonsson R. Predictors for work ability and disability in men and women with low-back or neck problems. Eur Spine J. 2006;15:780–793. 15937677 10.1007/s00586-004-0863-5 PMC3489465

[36] Mercieca-Bebber R, King MT, Calvert MJ, et al. The importance of patient-reported outcomes in clinical trials and strategies for future optimization. Patient Relat Outcome Meas. 2018;9:353–367. 30464666 10.2147/PROM.S156279 PMC6219423

