# Supplementary material for: Long-term effects on function, health-related quality of life and work ability after structured physiotherapy including a workplace intervention. A secondary analysis of a randomised controlled trial (WorkUp) in primary care for patients with neck and/or back pain
Source: Scand J Prim Health Care. 2020 Jan 30;38(1):92–100. doi: 10.1080/02813432.2020.1717081 (PMC7054906; doi:10.1080/02813432.2020.1717081)
Supplement: Supplemental Material [file IPRI_A_1717081_SM0324.docx]

**Supplementary material.**

Self-reported outcome measures by treatment group and by follow-up time*.

| **Outcome measure and follow-up month** | **Reference group** | | **Intervention group** | |
| --- | --- | --- | --- | --- |
|  | **Number of individuals** | **Mean (SD) / Median [IQR ]** | **Number of individuals** | **Mean (SD) / Median**  **[IQR]** |
| **Function (FRI)** |  | | | |
| 0 | 205 | 49.8 (18.7) | 145 | 46.5 (19.7) |
| 3 | 169 | 13.4 (10.7) | 124 | 13.3 (8.9) |
| 6 | 168 | 11.9 (8.0) | 121 | 10.7 (7.5) |
| 12 | 161 | 11.7 (8.2) | 112 | 10.5 (7.3) |
| **HRQoL (EQ-5D)** |  |  |  |  |
| 0 | 205 | 0.49 (0.30) | 144 | 0.53 (0.29) |
| 3 | 169 | 0.70 (0.23) | 123 | 0.67 (0.26) |
| 6 | 173 | 0.69 (0.25) | 123 | 0.72 (0.23) |
| 12 | 172 | 0.69 (0.27) | 115 | 0.74 (0.20) |
| **Work ability (WAS)** |  |  |  |  |
| 0 | 204 | 5.4 (2.9) / 6.0 [5.0] | 144 | 5.7 (2.6) / 6.5 [4.0] |
| 3 | 167 | 7.3 (2.3) / 8.0 [3.0] | 122 | 6.9 (2.3) / 7.0 [2.0] |
| 6 | 173 | 7.4 (2.2) / 8.0 [3.0] | 123 | 7.4 (1.9) / 8.0 [2.0] |
| 12 | 170 | 7.3 (2.4) / 8.0 [3.0] | 115 | 7.6 (2.1) / 8.0 [2.0] |

*****Unadjusted data
SD=standard deviation, IQR=interquartile range, FRI=Functional Rating Index, HRQoL=Health-related quality of life, EQ-5D=EuroQol five-dimension, WAS=Work Ability Score.
